# MicroRNA-17-5p promotes chemotherapeutic drug resistance and tumour metastasis of colorectal cancer by repressing PTEN expression

**DOI:** 10.18632/oncotarget.1614

**Published:** 2014-01-19

**Authors:** Lekun Fang, Haoran Li, Lei Wang, Jun Hu, Tianru Jin, Jianping Wang, Burton B Yang

**Affiliations:** ^1^ Guangdong Gastroenterology Institute, The Sixth Affiliated Hospital of Sun Yat-sen University, Guangzhou, China; ^2^ Sunnybrook Research Institute, Sunnybrook Health Sciences Centre, Toronto; ^3^ University Health Network, Toronto, Canada; ^4^ Department of Laboratory Medicine and Pathobiology, University of Toronto, Toronto

**Keywords:** microRNA, stem cell, miR-17, colon cancer, drug resistance

## Abstract

Background: Colorectal cancer (CRC) is one of the most common cancers worldwide, especially in Western countries. Although chemotherapy is used as an adjuvant or as a palliative treatment, drug resistance poses a great challenge. This study intended to identify biomarkers as predictive factors for chemotherapy.

Patients and methods: By microarray analysis, we studied miRNAs expression profiles in CRC patient, comparing chemoresistant and chemosensitive groups. The miRNAs of interest were validated and the impact on clinical outcomes was assessed in a cohort of 295 patients. To search for potential targets of these miRNAs, tissue samples were subject to *in situ* hybridization and immunohistochemistry analysis. Colorectal adenocarcinoma cells were also used for *in vitro* experimentation, where cellular invasiveness and drug resistance were examined in miRNA-transfected cells.

Results: The expression level of miRNA-17-5p was found increased in chemoresistant patients. Significantly higher expression levels of miR-17-5p were found in CRC patients with distant metastases and higher clinical stages. Kaplan-Meier analysis showed that CRC patients with higher levels of miR-17-5p had reduced survival, especially in patients who had previously received chemotherapy. Overexpression of miR-17-5p promoted COLO205 cell invasiveness. We found that PTEN was a target of miR-17-5p in the colon cancer cells, and their context-specific interactions were responsible for multiple drug-resistance. Chemotherapy was found to increase the expression levels of miR-17-5p, which further repressed PTEN levels, contributing to the development of chemo-resistance.

Conclusions: MiR-17-5p is a predictive factor for chemotherapy response and a prognostic factor for overall survival in CRC, which is due to its regulation of PTEN expression.

## INTRODUCTION

Colorectal cancer (CRC) is one of the leading causes of cancer mortality worldwide. It is estimated that over one million people develop colorectal cancer every year, especially in western countries [1]. Current guidelines recommend that treatments should be considered based on tumour stages. In potentially curable patients, surgery remains the mainstream treatment course, with or without adjuvant radiation and chemotherapy. In patients at advanced stages, palliative chemotherapy has been demonstrated to improve survival, by preventing tumour invasion or downsizing distant metastatic lesions. In the past three decades, the use of fluorouracil (5-FU), combined with irinotecan and oxaliplatin has been shown to double overall survival [2]. However, drug resistance poses a great challenge in treating chemorefractory patients. One of the most challenging tasks is to identify patient subpopulations that are most likely to respond to specific therapies. Therefore, understanding the mechanisms underlying chemoresistance may help identify subgroup of patients who may benefit from chemotherapy and avoid over-treatment. Despite enormous efforts, only a few predictive and prognostic biomarkers have been validated clinically [3]. Studies have shown that multiple cellular processes including DNA repair, cell apoptosis and proliferation may play important role in chemoresistance [4-6]. Several clinical studies have been performed in an attempt to find biomarkers predicting benefit from chemotherapy. However, with the exception of KRAS mutations, none of these studied markers have entered into the clinical management of colorectal cancer [7]. Given that complex signalling pathways and their cross-talk contribute to chemoresistance in a temporal- and spatial-specific manner, single molecular markers might not be sufficient to predict entire clinical outcomes. Thus, there is a great demanding to identify better markers that can enhance the prognostic strength in the clinical setting.

In recent years, microRNAs (miRNAs) have been recognized as key regulators of gene expression at the post-transcriptional level [8]. They are broadly involved in tumour proliferation, invasion, angiogenesis, and drug resistance [9-13]. High-frequency miRNA dysfunction is also associated with colorectal cancer development and progression [14]. It has been shown that miRNAs can be used as biomarkers for cancer detection [15]. One miRNA may be able to target several pathways, facilitating tumour cells evasion of drug treatment and generating stem-like cells [16]. Therefore, it is of value to illuminate whether dysregulation of these miRNAs-regulatory networks are also responsible for chemorefractory colorectal cancer. We identified miR-17-5p as a chemotherapy response predictor and prognostic biomarker in colorectal cancer. We found that miR-17-5p responded to chemotherapy by changing the levels of its target PTEN. Up-take of antisense oligo against miR-17-5p could successfully sensitize cancer cells to chemotherapy.

## RESULTS

### Expression of miR-17 in the course of colorectal cancer chemoresistance

**Fig 1:**
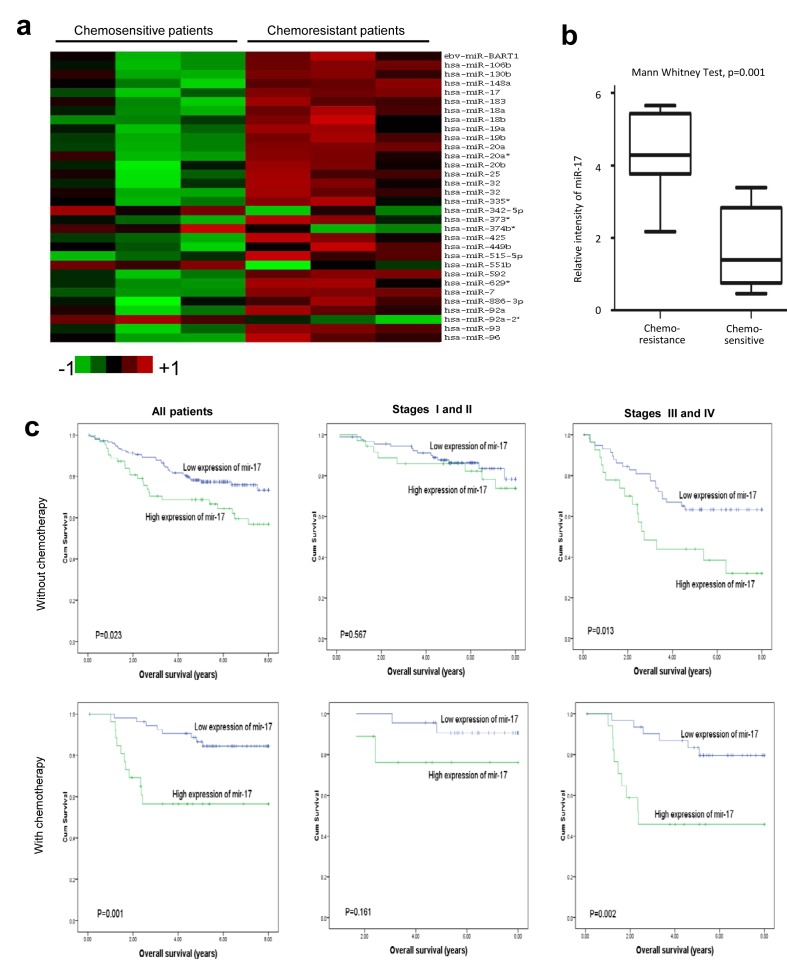
Expression of miR-17 is associated with chemoresistance in colorectal cancer **(a) Comparison of miRNA expression in three chemoresistant and three chemosensitive patient specimens by using the Agilent Human miRNA microarray.** Expression levels of miR-17-5p levels in chemoresistant patients were higher than those in chemosensitive patients. **(b) A validation experiment was carried out using qRT-PCR.** Expressions of miR-17-5p from fifteen primary colorectal cancer patients who consecutively underwent neoadjuvant chemotherapy were analyzed. Among them, seven patients were defined as complete response/partial response (CR/PR), while eight patients were defined as stable disease/progressive disease (SD/PD). Chemoresistant cancer samples have significantly higher levels of miR-17-5p (p=0.001, Mann Whitney Test). **(c) Association between miR-17-5p expression and overall survival in 81 patients with chemotherapy and 214 patients without chemotherapy.** High levels of miR-17-5p were associated with worse survival in colorectal patients, especially in those who received chemotherapy. For patients with cancer stages III and IV, high levels of miR-17-5p were significantly associated with poor survival, especially among those who received chemotherapy (P=0.002, Kaplan-Meier log rank test).

To search for potential miRNA targets in the course of colorectcal cancer (CRC) chemoresistance, we started with the analysis of miRNA expression profiles of CRC tissues collected before neoadjuvant chemotherapy. A comparison of miRNA expression levels between chemoresistant and chemosensitive groups are shown in Fig 1a. There were six miRNAs (miR-17-5p, miR-19b, miR-20a, miR-592, miR-7 and miR-93) showing consistently elevated levels in chemoresistant patients. Among them, miR-17-5p, miR-19b, miR-20a, miR-93 belonged to the miR-17~92 cluster and one of its paralogous clusters, miR-106b~25. Their overexpression has shown to be associated with many malignancies such as leukemia, liver and prostate cancer [17]. To confirm their potential roles in chemoresistance, validation experiments were carried out by qRT-PCR in seven chemoresistant and eight chemosensitive colorectal cancer samples. We found that the chemoresistant colorectal cancer samples had a significantly higher level of miR-17-5p than those obtained from chemosensitive colorectal cancer patients (Fig 1b, p=0.001, Mann Whitney test).

We further analyzed the association between miR-17-5p expression and therapeutic outcomes in CRC patients treated with adjuvant chemotherapy. The chemotherapy regimens were primarily fluorouracil-based, with leucovorin and oxaliplatin. Kaplan-Meier analysis demonstrated that high miR-17-5p expression was associated with a worse prognosis in CRC patients with chemotherapy (p=0.001), further indicating its potential as a predictive biomarker for chemotherapy (Fig 1c). Multivariate Cox regression showed increased miR-17-5p expression was predictive of a worse prognosis in CRC patients receiving chemotherapy (Table 1, HR 4.06, 95% CI 1.24 to 13.36, p=0.021). Therefore, miR-17-5p expression emerged as a predictive factor in the clinical outcomes of CRC patients treated with chemotherapy. We then analyzed association between miR-17-5p expression and survival in both early stage (Stage I and II) and late stage (Stage III and IV) CRC patients. A significant relationship between expression of miR-17-5p and overall survival rate in early stage CRC patients was not found. However, Kaplan-Meier analysis indicated that in late stage CRC patients, high miR-17-5p expression levels were associated with a worse prognosis, especially for patients who had received chemotherapy (Fig 1c).

**Table 1:** Univariate and multivariate analysis of different prognostic parameters in 81 colorectal cancer patients with chemotherapy

		Univariate analysis			Multivariate analysis	
	Variable	All cases	Mean survival (years)	p Value	HR (95% CI)	p Value
Sex	Male	45	7.026	0.091	1	0.019
	Female	36	6.122		4.371(1.272 to 15.020)	
Age	<58.8	48	6.668	0.896	1	0.292
	>58.8	33	6.563		1.826(0.595 to 5.601)	
Tumor location	Colon	43	7.166	0.032	1	0.019
	Rectum	38	6.002		4.085(1.264 to 13.199)	
Histological grade	G1-G2	59	6.652	0.991	1	0.573
	G3	22	6.539		0.711(0.217 to 2.331)	
pT status	T1-T2	6	6.987	0.803	1	0.999
	T3-T4	75	6.613		1.001(0.162 to6.184)	
pN status	N0	37	6.911	0.317	1	0.211
	N1	44	6.363		2.176(0.643 to 7.364)	
pM status	pM0	73	7.009	<0.001	1	<0.001
	pM1	8	3.147		20.494 (4.657 to 90.297)	
miR-17 expression	Low expression	55	7.286	0.001	1	0.021
	Overexpression	26	5.257		4.062 (1.235 to 13.355)	

**Table 2:** Univariate and multivariate analysis of different prognostic parameters in 295 patients with colorectal cancer

		Univariate analysis				Multivariate analysis	
	Variable	All cases	Mean survival (years)	P Value	HR (95% CI)		p Value
Sex	Male	153	6.638	0.493	1		0.359
	Female	142	6.352		1.236(0.786 to 1.944)		
Age	<58.8	134	6.694	0.158	1		0.080
	>58.8	161	6.340		1.523(0.951 to 2.439)		
Tumor location	Colon	147	6.841	0.022	1		0.004
	Rectum	148	6.170		2.040 (1.263 to 3.295)		
Chemotherapy	No	214	6.463	0.580	1		0.306
	Yes	81	6.621		0.748(0.429 to 1.304)		
Histological grade	G1-G2	249	6.596	0.193	1		0.498
	G3	46	5.982		1.227(0.679 to 2.216)		
pT status	T1-T2	49	6.924	0.415	1		0.869
	T3-T4	246	6.417		1.058(0.541 to 2.067)		
pN status	N0	181	6.795	0.018	1		<0.001
	N1	114	6.024		2.634(1.603 to 4.329)		
pM status	pM0	265	6.907	<0.001	1		<0.001
	pM1	30	2.942		11.68(6.513 to 21.0)		
miR-17 expression	Low expression	206	6.885	<0.001	1		0.007
	Overexpression	89	5.635		1.90(1.195 to 3.022)		

### MiR-17-5p induces drug resistance in colorectal cancer cells

**Fig 2:**
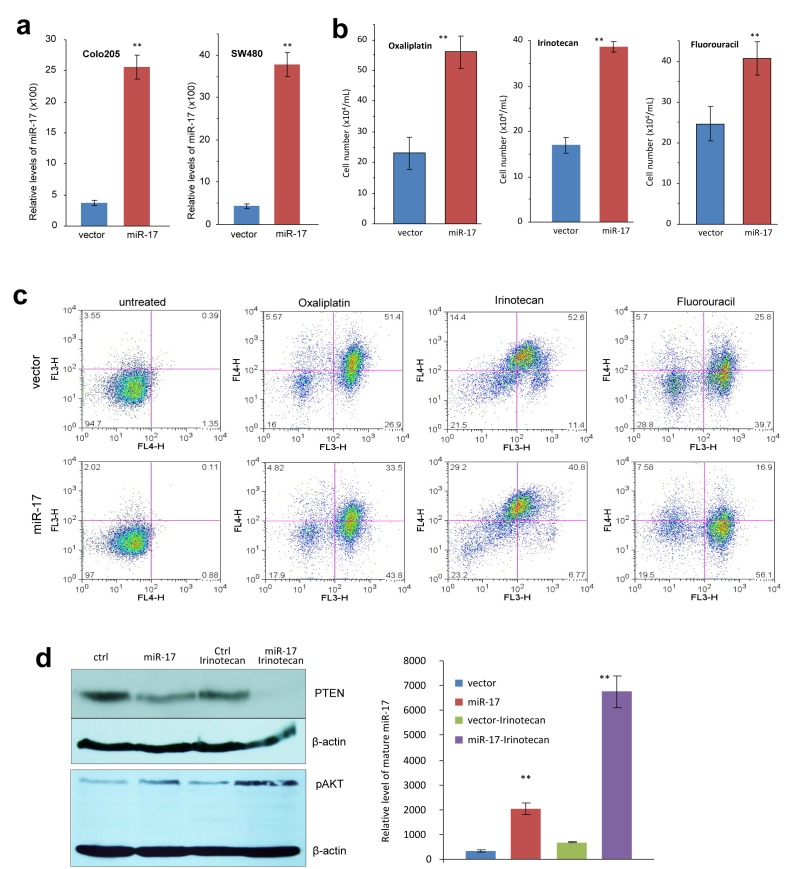
MiR-17 induces multiple drug resistance in colorectal adenocarcinoma cells **(a) Real-time PCR was performed to measure miR-17-5p levels in COLO205 and SW480 cells transfected with the control vector or miR-17.** Increased RNA levels were observed in the miR-17-transfected cells compared to vector control. **(b) COLO205 cells were treated with Oxaliplatin, Irinotecan and fluorouracil (5-FU) overnight, followed by counting cell number.** More cells survived in miR-17 overexpression group. **p<0.001, Error bars indicate SD, n=3. **(c) COLO205 cells were treated with Oxaliplatin, Irinotecan and fluorouracil overnight, followed by analysis of apoptosis.** There were fewer cells undergoing apoptosis in the miR-17 overexpression group. **(d) Left, COLO205 cells were cultured in medium with or without Irinotecan** Cell lysate prepared was analyzed on Western blot for expression of PTEN and pAKT. While cells transfected with miR-17 expressed lower level of PTEN than the control, treatment with Irinotecan further decreased PTEN levels, especially in cells overexpressing miR-17. Staining of β-actin from the same membrane confirmed equal loading. On the other hand, expression of pAKT was opposite. Right, Irinotecan treatment increased miR-17-5p levels, especially in the cells transfected with miR-17 overexpression plasmid.

To initiate the dissection of the function of miR-17-5p in colorectal tumorigenesis, we conducted the *in vitro* investigations. We stably transfected a miR-17 overexpression plasmid and its control vector expressing a non-related sequence into colorectal cancer cell lines COLO205 and SW480. The construct we developed contained a pair of human pre-miR-17 units, which were used to generate over-expression of mature miR-17-5p (Supplementary Fig S1a). Real-time PCR was used to verify increased levels of miR-17-5p in the transfected cells, as compared with the control cell lines (Fig 2a). As mentioned above, miR-17-5p is negatively related with chemosensitive status in CRC patients. Based on MTT assay, we applied cytotoxic drugs (Oxaliplatin, Irinotecan, and Fluorouracil) at the half maximal inhibitory concentration (IC50) to cultured COLO205 cells. After 12 hour treatment, miR-17-transfected cells showed greater resistance towards these chemotherapeutic agents, with more cells surviving after the treatment (Fig 2b and Supplementary Fig S1b). We then conducted an apoptosis assay to verify our findings through flow cytometry and found that miR-17 overexpression decreased cellular apoptosis induced by chemotherapeutic treatments (Fig 2c).

In a previous study, we found that the loss of PTEN resulted in activation of downstream signaling pathways, which accounted for the drug resistance observed in cancer cells [16]. To trace the change of PTEN during the course of chemotherapy, we analyzed the levels of PTEN expression by Western blotting. Although PTEN was down-regulated in the miR-17-transfected cells before Irinotecan treatment, a much more drastic decrease was observed following Irinotecan treatment (Fig 2d, left). Consistent with these results was the up-regulation of pAKT. We also found a concomitant up-regulation of miR-17-5p, which was substantially increased in response to chemotherapeutic treatment (Fig 2d, right). It appears that targeting of PTEN by endogenous miR-17-5p became a prominent factor in cellular stress induced by the chemotherapeutic regimens. We hypothesize that miR-17-5p is a central mediator of chemoresistance, enabling colorectal cancer cells to escape chemotherapy.

### PTEN as a target of miR-17-5p in colorectal cancer cells

**Fig 3:**
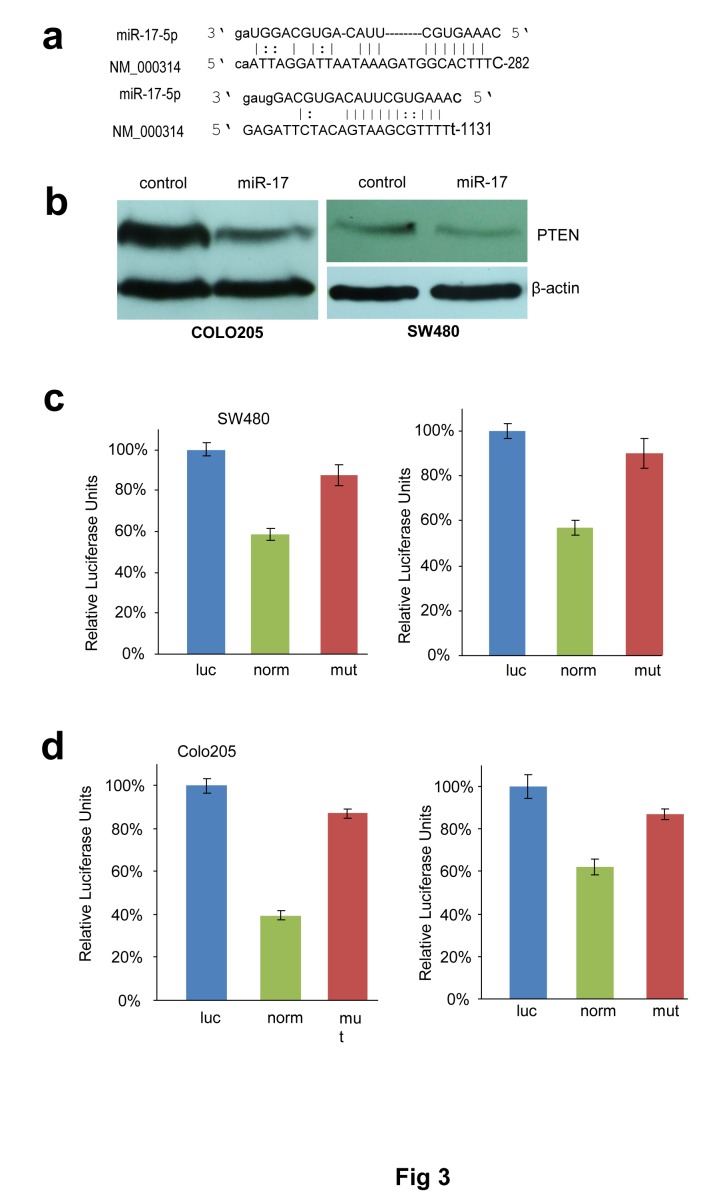
PTEN is targeted by miR-17-5p in colorectal adenocarcinoma cells (a) Computational analysis showing that miR-17-5p potentially targeted PTEN at two different sites. (b) Cell lysates prepared from miR-17- or mock-transfected COLO205 and SW480 cells was analyzed on Western blot, showing repression of PTEN expression in the miR-17-transfected cells. **(c) SW480 cells were co-transfected with miR-17 and each of the luciferase reporter constructs or the mutants.** The luciferase reporter vectors (Luc) were used as controls. **(d) COLO205 cells were co-transfected with miR-17 and each of the luciferase reporter constructs or the mutants.** The luciferase reporter vectors (Luc) were used as controls. MiR-17-5p repressed the activity of Luc-Pten-1 and Luc-Pten-2 but had no effect on that of Luc-Pten-1mut and Luc-Pten-2mut. Error bars, SD (n=3).

PTEN is a tumour suppressor which dominates the PTEN/AKT/PI3K pathway. Loss of PTEN and activation of AKT has been reported in many types of cancers, including hepatocellular carcinoma, prostate adenoma and colorectal cancer [18]. Through computational analysis, we found that the 3'-untranslated region of PTEN mRNA contained two binding sites for miR-17-5p (Fig 3a). Western blot analysis was thereby performed and PTEN was to be found decreased in miR-17-transfected cells (Fig 3b). We then generated firefly luciferase reporter constructs with the 3'UTR of PTEN mRNA, and transfected them into colorectal cancer cells with miR-17-5p mimics. We found that co-transfection with miR-17-5p in SW480 and COLO205 cells decreased luciferase activity when the construct contained the 3'UTR of PTEN (Fig 3c, Fig 3d). Mutation of the binding sites reversed the observed inhibitory effects.

**Fig 4:**
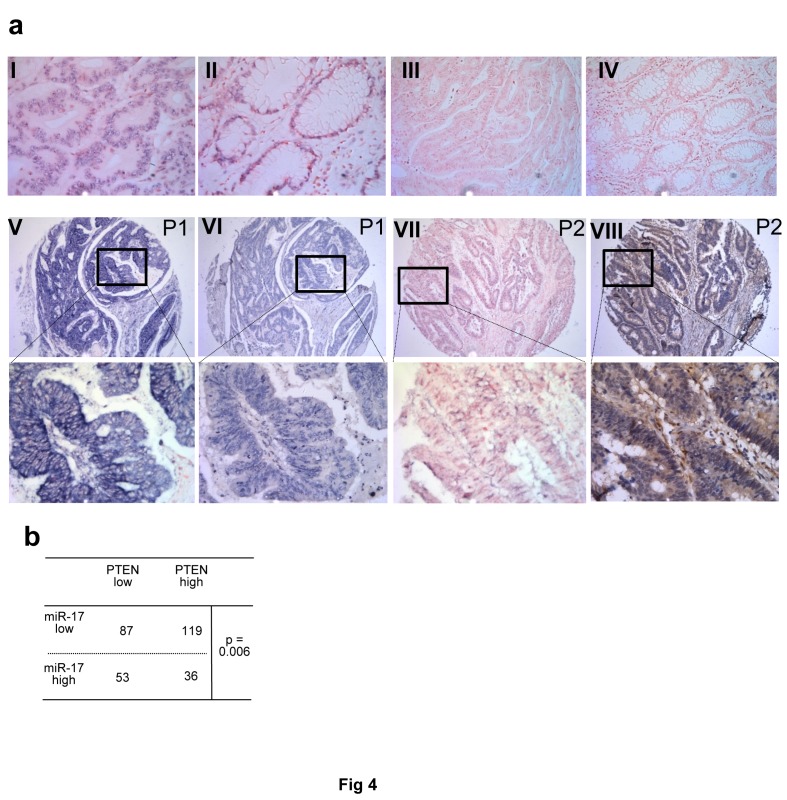
PTEN expression is negatively associated with miR-17-5 level in colorectal tissue **(a) In Situ Hybridization (ISH) of miR-17-5p expression and immunohistochemistry (IHC) of PTEN in colorectal cancer specimens: tissue sections were incubated with a full length DIG-labelled LNA probe to miR-17-5p for ISH or the first antibody to PTEN for IHC.** The positive ISH staining was expressed as blue-violet and the positive IHC staining was brown. (I) Staining of U6 in cancer sample (positive control). (II) Staining of U6 in normal colon tissue. (III) Staining of scramble control probe in cancer sample (negative control). (IV) Staining of scramble control in normal colon tissue. (V and VII) Representative staining of miR-17-5p in patient 1 (P1 showing high level of miR-17-5p) and patient 2 (P2 showing low level of miR-17-5p). (VI and VIII) Representative staining of PTEN in patients 1 and 2. Top panel:×200; middle panel: ×100. **(b) Mir-17-5p expression was inversely associated with expression of PTEN.** P value was calculated by Pearson Chi-square test.

Next we conducted In Situ Hybridization (ISH) assays to detect miR-17-5p expression in colorectal cancer tissues. PTEN expression was also analyzed by immunohistochemistry (IHC) in these samples (Fig 4a). In cancer tissues where miR-17-5p was overexpressed (Fig 4aV), PTEN was down-regulated (Fig 4aVI). Consistent with this, low expression of miR-17-5p was correlated with high PTEN expression (Fig 4aVII vs. Fig 4aVIII). We further validated the association between miR-17-5p and PTEN expression levels in 295 colorectal cancer specimens. miR-17-5p was found elevated in 89 samples, 53 of which showed reduced expression levels of PTEN. By Pearson Chi-square test, it was shown that miR-17-5p was inversely correlated with PTEN expression (*p*=0.006) (Fig 4b).

**Fig 5:**
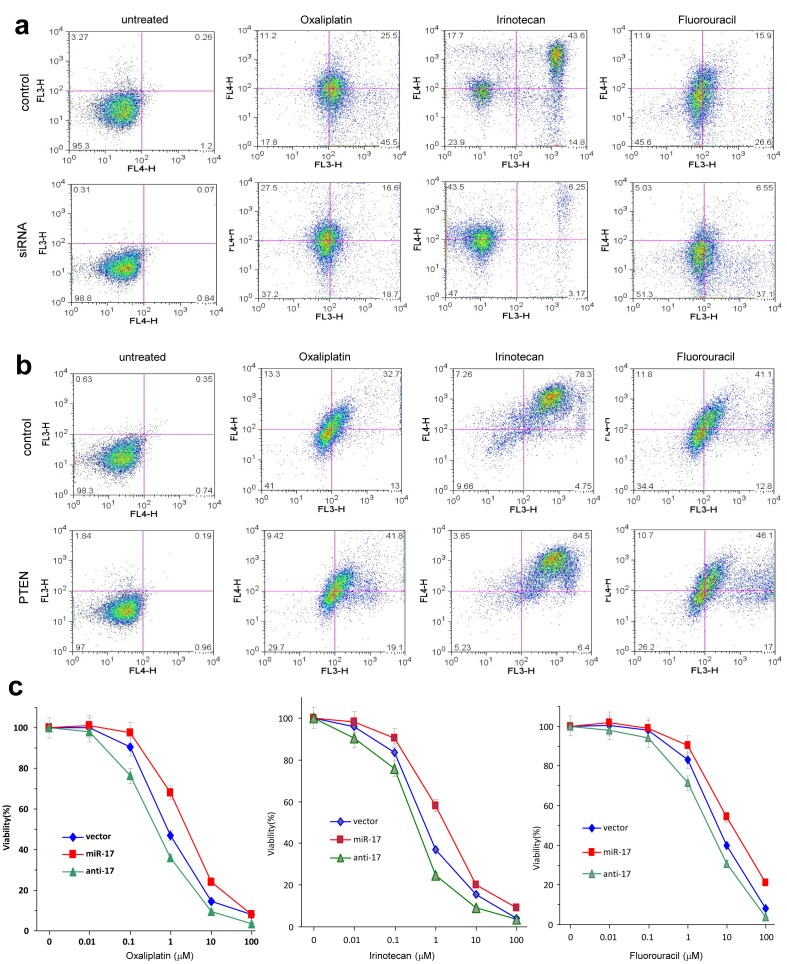
MiR-17-5p promotes multiple drug resistance by regulating PTEN **(a) COLO205 cells transiently transfected with siRNA targeting PTEN or a control oligo were subject to apoptosis assay after cytotoxic drug treatment.** Down-regulation of PTEN decreased cell sensitivity to multiple chemotherapeutic agents. **(b) COLO205 cells stably transfected with miR-17 were transiently transfected with PTEN expression construct or the control vector.** Up-regulation of PTEN increased cell sensitivity to multiple chemotherapeutic agents. **(c) The miR-17-, vector- and antisense oligonucleotides-transfected COLO205 cells were cultured and treated with Oxaliplatin, Irinotecan, and Fluorouracil, followed by MTT analysis of cellular viability.** Cells transfected with miR-17 displayed resistance to all drugs, yet anti-miR-17-5p treatment arrested miR-17-5p's function. Error bars, SD (n=3).

We then tested whether PTEN mediated the survival effects observed in cancer cells treated by chemotherapy. When treated with siRNA against PTEN, more cells survived after chemotherapeutic treatment (Fig 5a). More importantly, reconstruction of PTEN expression sensitized these cells to cytotoxic drugs, with more cells undergoing apoptosis and cell death (Fig 5b). By complementary binding to miRNA, antagomir or antisense small RNAs can arrest miRNA's functioning by preventing further processing [19]. When we transiently transfected antisense oligos against miR-17-5p into COLO205 cells, we found that the cells became more sensitive to drug treatment than control cells (Fig 5c). Increased drug sensitivity was observed in anti-miR-17-transfected cells, co-cultured overnight with fluorouracil, irinotecan and oxaliplatin. These results suggested that miR-17 could be a therapeutic target in the treatment of chemorefractory colorectal cancer.

### Relationship between mir-17-5p expression and overall survival of CRC patients

**Fig 6:**
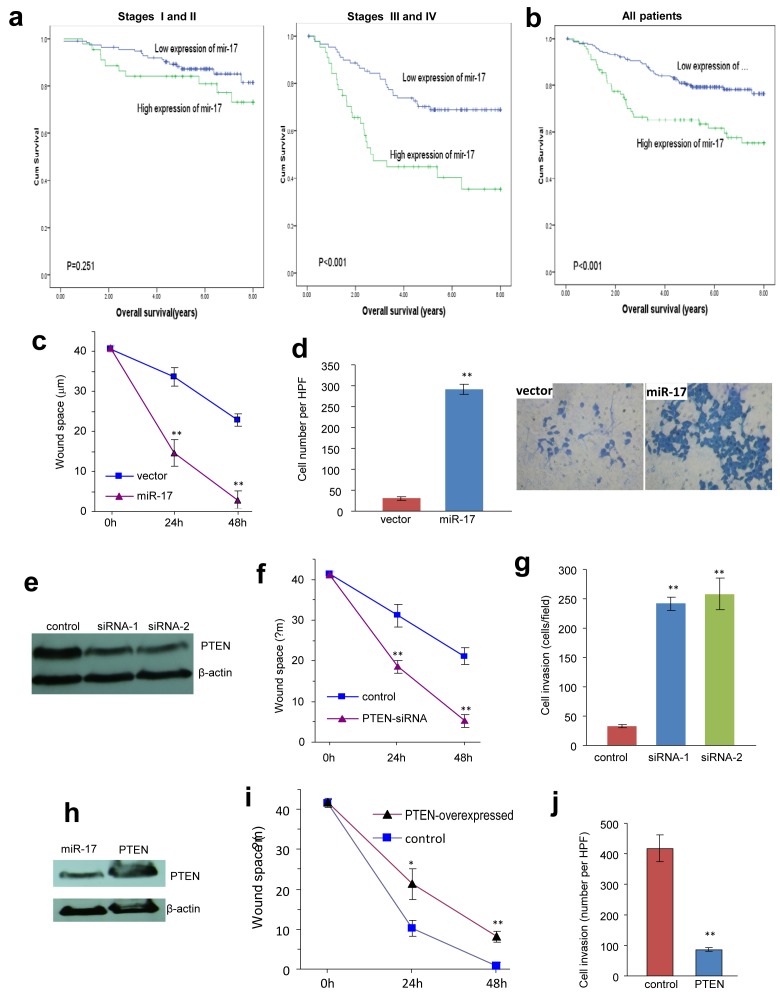
Overexpression of miR-17-5p is associated with increased tumour metastasis and poor survival **(a) Among the patients with stage I and II, there is no statistically significant association between miR-17-5p expression and prognosis (p>0.05, Kaplan-Meier log rank test).** However, high levels of miR-17-5p was significantly associated with worse prognosis in patients with stage III and IV disease (p<0.001, Kaplan-Meier log rank test). **(b) Association between miR-17-5p expression in colorectal cancers and overall survival in 295 patients with colorectal cancer.** For all of the colorectal patients, Kaplan-Meier test showed that high miR-17-5p expression associated with poor overall survival (P<0.001, Kaplan-Meier log rank test). **(c) The miR-17- and vector-transfected cells were seeded onto 6-well dishes.** The monolayers were wounded with a pipette tip and cultured in 10% FBS/DMEM medium containing 2 μM mytomycin. The distances between the wounding centre and the front of the migrating cells (vertical axis) were measured for statistical analysis. **, p<0.01. Error bars indicate SD (n=12). **(d) The cells were loaded into the insert with 100 μL serum-free DMEM medium and then incubated at 37°C for 24 hours.** The invasive cells were stained blue and were counted in 6 randomly selected fields under a light microscope. Expression of miR-17-5p promoted cell invasion. **, p<0.01. Error bars indicate SD (n=6). **(e) Cell lysates prepared from COLO205 cells transiently transfected with siRNA targeting PTEN or a control oligo were subject to Western blot analysis probed with anti-PTEN antibody to confirm silencing of PTEN.** **, p<0.01. Error bars indicate SD (n=12). **(f) Wound scratch assay showed that cells transfected with siRNA silencing PTEN migrated faster than control.** **, p<0.01. Error bars indicate SD (n=6). **(g) Trans-well invasion assay showed that down-regulation of PTEN promoted cell invasion.** **, p<0.01. Error bars indicate SD (n=12). **(h) Cell lysate prepared from cells transiently transfected with PTEN expression construct or the control vector and subject to Western blot analysis probed with anti-PTEN antibody to confirm expression of the construct.** Staining of β-actin from the same membrane confirmed equal loading. **(i) Wound scratch assay showed that overexpression of PTEN retarded cell migration.** **, p<0.01. Error bars indicate SD (n=12). **(j) Trans-well invasion assay showed that up-regulation of PTEN inhibited cell invasion.** **, p<0.01. Error bars indicate SD (n=6).

We assessed the impact of mir-17 expression on overall survival in a patient cohort. The clinicopathological characteristics of the CRC patients are summarized in Supplementary Table S1. The testing cohort consisted of 153 men and 142 women, with a total of 81 CRC patients, who were treated by adjuvant chemotherapy. High expression levels of miR-17-5p were found in 89/295 (30.17%) of patients. Significantly higher miR-17-5p expression levels were found in CRC patients with distant metastasis and higher clinical stages (Fig 6a, Table S1). Kaplan-Meier analysis showed that N status, distant metastasis, clinical stage and miR-17-5p expression were correlated with poor overall survival (Table 2). CRC patients with high expression levels of miR-17-5p had reduced survival than patients with low expression levels of miR-17-5p (p=0.001, log-rank test, Fig 6b). Further multivariate Cox regression analysis determined that tumor location, N status, distant metastasis and expression of miR-17-5p were independent prognostic factors for the poor survival of CRC patents (Table 2). The results also demonstrated that there was no significant association between miR-17-5p expression and other clinicopathological features, such as patient gender, age, tumor location, T classification, N classification and chemotherapy.

### Mir-17-5p promoted colorectal cancer cell migration

Previous studies have shown that miR-17 overexpression is related to tumour cell growth [20]. In our study, we found that neither cell survival (Fig S1c) nor cell proliferation (Fig S1d) was altered by miR-17 transfection. However, scratch wound assays performed on cancer cell monolayers revealed that the miR-17-transfected cells migrated faster than the control cells (Fig 6c, Fig S2a). Moreover, when seeded on the upper chamber of trans-well plates, more cells in the miR-17 group were able to migrate through to the other side of the chamber (Fig 6d, Fig S2b). Taken together, the miR-17-transfected colon cancer cells showed greater motility in culture conditions, which suggested higher metastatic potential *in vivo*. These results were in line with our clinical findings, showing that patients with metastatic disease had higher expression levels of miR-17-5p (Table S2).

To validate miR-17's function in colorectal cancer cells, we employed siRNAs against PTEN to simulate miR-17-5p overexpression (Fig 6e). Down-regulation of PTEN would result in activation of the AKT/PI3K/HIF-1α pathway, which contributed to cancer cell migration [21]. As expected, we detected increased cell motility (Fig 6f) and cell invasion (Fig 6g, Fig S2b) in the siRNA treated cells.

To corroborate these results and counteract miR-17's effect, we overexpressed PTEN in COLO205 cells (Fig 6h). As a consequence, we detected decreased cell migration in the PTEN-overexpressing group by both scratch wound (Fig 6i, Fig S2c) and trans-well migration assays (Fig 6j, Fig S2d). Therefore, it was confirmed that miR-17-5p enhanced invasiveness of colorectal cancer cells by targeting the PTEN pathway. The addition of cytotoxic drugs in culture media beforehand was not found to counteract these aggressive migration phenotypes (Data not shown). Given that increased motility is often associated with higher metastasic capacity [22], our data suggests that miR-17-5p promoted colorectal cancer cell metastasis in a treatment-independent manner. In 81 colorectal patients with chemotherapy and 214 patients without chemotherapy, miR-17-5p overexpression was found to be predictive of worse overall survival (Tables 1, Table S2). Given our in vitro and in vivo results, we concluded that miR-17 overexpression contributed to tumour metastasis, leading to decreased overall survival in CRC patients.

## DISCUSSION

Currently, fluorouracil based chemotherapy remains a standard treatment course for patients with advanced CRC. While improving patient survival and reducing recurrence, chemotherapy resistance leading to treatment failure and local recurrence is still a critical problem. One of the biggest challenges is to identify patient subpopulations that are most likely to respond to specific therapies. If one or more biomarkers could predict patient's response to chemotherapy, we could more effectively treat these patients, while redirecting other groups to alternative strategies that could be more effective. Considering that the poor prognosis in CRC patients is typically due to late diagnosis and low chemotherapy response, it is of importance to identify predictive markers of therapeutic response.

In the present study, microRNA expression profile was first examined in CRC samples from patients who received neoadjuvant chemotherapy. We discovered that miR-17-5p was capable of conferring a responder or nonresponder status in colorectal cancer patient samples. Further results showed that miR-17-5p was an independent predictive factor in patients who received chemotherapy. We also demonstrated that miR-17-5p might induce chemoresistance by regulating PTEN expression. In CRC samples, the expression levels of miR-17-5p were found to be correlated inversely with PTEN expression. Subsequent analysis indicated that miR-17-5p was significantly correlated with tumour metastasis and advanced clinical stage. This suggested that the overexpression of miR-17-5p in CRC may facilitate the invasive/metastatic phenotype. Taken together, overexpression of miR-17-5p in CRC was a strong and independent predictor of chemotherapy response and a prognostic biomarker for worse survival. Examination of miR-17-5p expression levels could be used as an additional tool in identifying CRC patients who are in need of chemotherapy or are at a risk of tumor metastasis.

Resistance to chemotherapy may arise from inherent genetic instability or through selection of environmental stress. Recently, miRNAs have emerged as crucial mediators in regulating the cellular responses of cancer cells to therapy. Patient response to chemotherapy has shown to be closely correlated to the functional status of microRNAs [23-25]. Although the mechanisms of miRNA-regulated drug resistance are still largely unknown, current evidence suggest several roles for miRNA, including influence of therapeutic induced cell death, alteration of drug targets, regulation of multiple drug resistance (MDR)-related proteins, change in bioavailable drug concentration and promotion of angiogenesis and tumor stem-like cells (TSC) [10]. Some miRNAs are capable of conferring drug resistance by targeting PTEN. For example, it has been reported that miR-214 induces cell survival and chemoresistance, by binding the 3'UTR of PTEN mRNA [26]. In our previous study, we found that miR-17-5p targeted an oncogene, MDM2 and a tumor suppressor PTEN simultaneously, resulting in chemoresistance and generation of TSCs in glioblastoma [16]. Loss of PTEN is a very frequent genetic aberration in malignant tumours such as breast cancer, gastric cancer and glioblastoma. Various studies have suggested that PTEN loss is significantly associated with cytotoxic drug resistance [27, 28].

In this study, we found that miR-17-5p negatively regulated PTEN expression in colorectal carcinoma cell lines COLO205 and SW480. As a result, these cells became more aggressive and invasive after transfected with the miR-17 expression construct. Experiments *in vitro* showed that the miR-17-transfected cells migrated faster than control cells in both two- and three- dimensional environments, which could be linked to more distant metastasis *in vivo*. This finding is consistent with clinical observations, which revealed that more advanced patients expressed higher levels of miR-17-5p. There is growing evidence suggesting that dysfunction of PTEN has prognostic importance in several malignancies, including colorectal cancer [29]. Our findings reveal that targeting PTEN at the post-transcriptional level by miRNAs such as miR-17-5p are also responsible for PTEN inactivation, and are thereby associated with reduced survival in CRC patients. PTEN down-regulation is closely correlated with PI3K/Akt activation, and this cascade pathway has profound effects on tumorigenesis, proliferation, migration, and apoptosis. We did not observe a difference in cell growth, which could be due to minimal repression of PTEN translation in normal conditions. Interestingly, we found that miR-17-5p levels were elevated upon chemotherapeutic stress, leading to increased knock-down of PTEN. As a result, cells overexpressing miR-17-5p survived better than the controls. PTEN exerts an essential role in maintaining chromosomal integrity and cell cycle progression [30]. In response to DNA damage, cancer cells often activate PI3K/Akt pathway, which modulate cell survival signalling and regulate DNA repair machinery directly [31]. Moreover, inactivation of PTEN also has a positive effect on cancer cell proliferation, which can contribute to therapeutic resistance and tumour recurrence [32]. Our studies suggest that the miRNA-regulatory network might be the first responder in face of DNA damaging signalling, and overexpression of miRNAs trigger various response cascades for cell survival.

In summary, we identified miR-17-5p as a chemotherapy response predictor and prognostic biomarker in colorectal cancer. Furthermore, we found that miR-17-5p responded to chemotherapy by changing the levels of both itself and its target, PTEN. Up-take of antisense oligo against miR-17-5p could successfully sensitize cancer cells to chemotherapy. Future therapeutic strategies could be developed based on the predictive levels of miR-17-5p. Surgery, instead of chemotherapy, might therefore be more suitable for colorectal cancer patients with high levels of miR-17-5p expression. In future, strategy could be developed to repress miR-17-5p levels combined with chemotherapy.

## MATERIALS AND METHODS

### Patients

Fifteen patients with primary colorectal cancer who consecutively underwent neoadjuvant chemotherapy containing fluorouracil (5-FU) at the Department of Colorectal Surgery, the Sixth Affiliated Hospital of Sun Yat-Sen University, were enrolled into the present study. Tumor specimens were obtained by colonoscopy prior to starting therapy. The effect of chemotherapy on the tumors was assessed as the three-dimensional volume reduction rate or tumor response rate. The tumor response was evaluated by the Response Evaluation Criteria in Solid Tumors (RECIST), which is defined as the following: complete response (CR; disappearance of the disease), partial response (PR; reduction of ≥30%), stable disease (SD; reduction <30% or enlargement ≤20%), or progressive disease (PD; enlargement ≥20%). Among them, 7 patients were defined CR/PR, and 8 patients were defined SD/PD.

Paraffin-embedded samples of primary colorectal adenocarcinomas were included from 295 patients, who underwent tumor resection between 2001 and 2005 at the First Affiliated Hospital of Sun Yat-Sen University. This cohort of patients with CRC included 153 (51.9%) men and 142 (48.1%) women, with a median age of 59 years, and their clinic-pathological characteristics are summarized in Supplementary Table S1. The cases selected were based on a distinctive pathological diagnosis of CRC, undergoing primary and curative resection for CRC, availability of resection tissue, follow-up data, and had not received preoperative anticancer treatment. Our study protocol was approved by The Ethics Committee of the First and the Sixth Affiliated Hospital.

### Microarray

Total RNAs were extracted from tissues of six primary colorectal cancer patients using the mirVana miRNA extraction kit (Ambion) according to the manufacturer's instructions. The quality control of RNA was performed by a 2100 Bioanalyzer using the RNA 6000 Pico LabChip kit (Agilent Technologies, Santa Clara, CA). The microarray was performed at the Shanghai Biochip Company by using the Agilent Human miRNA microarray Kit version 12.0. Total RNA (100 ng) derived from each of the specimens were used as inputs for labelling via Cy3 incorporation. Microarray slides were scanned by XDR Scan (PMT100, PMT5). The labelling and hybridization were performed according to the protocols in the Agilent miRNA microarray system.

### RNA isolation and quantification of miRNA by qRT-PCR

RNA samples were isolated from harvested cells using Trizol reagent (Invitrogen) according to the manufacturer's instructions. miRNA expression was quantified by two-step quantitative RT-PCR, beginning with first-strand cDNA synthesis using the One-step primeScript miRNA cDNA Synthesis Kit (Takara), followed by quantitative real-time PCR using the miRscript SYBR Green PCR kit in a 7500 Real-Time PCR system. The mature miRNA-specific forward primer was purchased from Takara (DHM0136) and the universal reverse primer was provided by the manufacturer. RNA quantity was normalized using U6 snRNA, and fold change of expression was calculated according to the 2^−ΔΔct^ method.

### Tissue Microarrays

The tissue microarray (TMA) was conducted using paraffin-embedded tissues. In brief, the paraffin-embedded tissue blocks and the corresponding histological H&E stained slides were overlaid for tissue TMA sampling. Duplicate of 0.6 mm diameter cylinders were punched from representative tumor areas of individual donor tissue block, and re-embedded into a recipient paraffin block at a defined position, using a tissue arraying instrument (Beecher Instruments, Silver Spring, MD).

### In situ hybridization and Immunohistochemistry

For in situ hybridization, tissue slides were deparaffinized and digested with proteinase K for 30 min. The slides were then prehybridized in a hybridization solution at 570C for 2 hours. Ten picomoles of digoxingenin-labeled miRCURY LNA detection probes (Exiqon) complementary to U6 or miR-17-5p or scrambled microRNA were added and hybridized at 550C for 1 hour. After stringent washes, an immunologic reaction was carried out by using the biotinylated sheep antibody against digoxingenin (Roche) and with alkaline phosphatase streptavidin (Zhongshan Golden Bridge Biotechnology Company) to detect biotinylated probes.

For immunohistochemistry, the paraffin sections were incubated with primary antibody against PTEN (1:100, CST, USA). For negative control, isotype-matched antibodies were applied. Each slide was assigned a score for density and intensity. Slides were mounted with mounting medium and analyzed using a Leica DMI4000B microscope. Each slide was assigned a score for intensity and staining positive pattern.

The percentage of positive tumor cells was set as follows: 1 (up to 25% of positive cells), 2 (25% to 50% of positive cells), 3 (50% to 75% of positive cells) and 4 (more than 75% of positive cells). Intensity scores ranged from 0-3: 0, no staining; 1, weak staining; 2, moderate staining, and 3, strong staining. Multiplication of the two scores resulted in a final score ranging from 0 to 12. Under these conditions, samples with score 0-6 and score 8-12 were defined as low and high expression.

### Cell cultures

Human colorectal adenocarcinoma cell lines COLO205 (CCL-222) and SW480 (CCL-228) were cultured in Dulbecco's Modified Eagle's Medium (DMEM) supplemented with 10% fetal bovine serum (FBS), penicillin (100 U/mL) and streptomycin (100 U/mL). Cells were allowed to grow in a humidified incubator containing 5% CO_2_ at 37°C and subcultured every 3-4 days.

### Construct generation

We generated a cDNA sequence which contains a pair of human pre-miR-17 units, a CMV promoter driving expression of green fluorescence protein (GFP) and an H1 promoter. It was then inserted into an expression vector pEGFP-N1 between the restriction sites BglII and HindIII. Successful transfected cells were screened by using green fluorescence and cultured in media with G418 at the concentration of 1 mg/ml.

For luciferase assay, computational analysis showed two potential binding sites for miR-17-5p in the 3'-untranslated region (3'UTR) of PTEN. Thus, two pairs of primers were used to clone the fragments as well as mutant controls. The PCR products were then digested with SacI and MluI, followed by insertion into a SacI- and MluI-digested pMir-Report vector (Ambion) to obtain a luciferase construct or a mutant counterpart. In PTEN rescue test, the PTEN cDNA with coding region was purchased from Origene.

### Real-time PCR analysis

For real-time PCR analysis, total cellular RNA was extracted by using the mirVana miRNA Isolation Kit (Ambion) according to the manufacturer's instructions. The cDNA products were synthesized by using 1 μg RNA in successive reverse transcription PCR, which was performed using miScript Reverse Transcription Kit (Qiagen). The primers specific for mature miR-17-5p were purchased from Qiagen and real-time PCR was performed by using miScript SYBR GreenPCR Kit (Qiagen). The primers used as controls for real-time PCR were Human-U6RNA.

### Cell activity tests

In cell proliferation assay, transfected COLO205 and SW480 cells were plated onto 12-well tissue culture plates at a density of 1×10^5^ cells/well for 5 days. Meanwhile, survival assay was performed by keeping the cells (1×10^6^ cells/well) in serum-free medium for 10 days. The cells were harvested and cell number was counted in different time points.

To test drug sensitivity, fluorouracil (Valeant Pharmaceuticals), eloxatin (Oxaliplatin) (Sanofi-Aventis) and irinotecan (Pfizer) were applied to adhered cell cultures. These drugs were purchased from the Pharmacy Department at Sunnybrook Health Sciences Centre. The cell number was counted 12 hours after drug loading to the cultures by Trypan Blue staining. They were also subjected to apoptosis assay by flow cytometry.

For wound scratch assay, monolayer of cells was scraped linearly with micropipette tips (BioMart) and washed to remove cell debris. To diminish the impact of proliferation, the cultures were treated with Mitomycin C (Sigma) at 200 µg/mL for two hours beforehand. Microscopic images were captured at the beginning, 24 hours and 48 hours intervals, and the migrated distance was quantified. In the transwell invasion assay, 24-transwell (Coster) was coated with 100 μL BD MatrigelTM (BD Biosciences). COLO205 cells at a density of 1×10^5^/100 μL were suspended in DMEM media and transferred into the upper chamber of the transwell. The lower chamber was filled with 600 μL DMEM media containing 10% FBS. After incubation for 12 hours, non-migrated cells were removed with cotton swab and invaded cells were stained with Coomassie brilliant blue (Bio-Rad).

### Western blot

In Western blot analysis, cell lysates were collected from the cultured cells, which were subject to sodium dodecyl sulfate polyacrylamide gel electrophoresis (SDS-PAGE). The proteins separated on SDS-PAGE were transferred onto a nitrocellulose membrane (Bio-Rad). The membrane was then blocked in Tris-Buffered Saline and Tween 20 (TBST: 10 mM Tris-Cl, 150 mM NaCl and 0.05% Tween 20) containing 10% skim milk powder for 30 minutes. It was then incubated at 4overnight with mouse monoclonal anti-PTEN antibody (Abcam). After washing for 30 minutes, secondary goat anti-mouse IgG (Vector) was applied to nitrocellulose membrane in TBST for 1 hour. After washing for 1 hour, the proteins of interest were visualized by using Chemiluminescent HRP Antibody Detection Kit (Denville Scientific).

### Flow cytometry

For cell cycle analysis, cells in the logarithmic phase of growth were harvested and washed twice in PBS. Following adjustment of a cell concentration to 2×10^6^ cells/mL in 50 µL PBS/HBSS with 2% calf serum, 1 mL 80% ice cold ethanol was added and incubated for 30 minutes. The cells were re-suspended in 500 µL HBSS containing 0.1 mg/mL of Propidium Iodide (Sigma) and 0.6% of NP-40. The DNA content was measured by flow cytometry (Beckman Coulter).

Cell apoptosis was detected by using Annexin V-FITC Apoptosis Detection Kit (BD Pharmingen). According to the manufacture's instruction, 1×10^6^ cells were washed twice in PBS before re-suspension in 50 µL HBSS with 2% calf serum. Annexin V-FITC and Propidium Iodide of 5 µL respectively were added and stained on ice for 30 minutes. The cells were re-suspended to 500 µL HBSS and flow cytometry analysis was conducted within 30 minutes. The data were analyzed using FlowJo9.1 software.

### Luciferase activity assay

Luciferase activity assays were performed as previously described [16]. COLO205 and SW480 cells were seeded onto 12-well tissue culture dishes at a density of 1×10^5^ cells/well and co-transfected with the luciferase reporter constructs and miR-17-5p mimic with Lipofectamine 2000. After 12 hours, cell lysate was prepared by using Dual-Luciferase® Reporter Assay Kit (Promega) and luciferase activity was detected by microplate luminescence counter (Perkin Elmer).

## SUPPLEMENTARY FIGURES AND TABLES




