# Supplementary material for: MicroRNA-17-5p promotes chemotherapeutic drug resistance and tumour metastasis of colorectal cancer by repressing PTEN expression
Source: Oncotarget. 2014 Jan 19;5(10):2974–87. doi: 10.18632/oncotarget.1614 (PMC4102784; doi:10.18632/oncotarget.1614)
Supplement: Supplementary file 1 [file oncotarget-05-2974-s001.pdf]

MicroRNA-17-5p promotes chemotherapeutic drug resistance and tumour metastasis of colorectal cancer by repressing PTEN expression

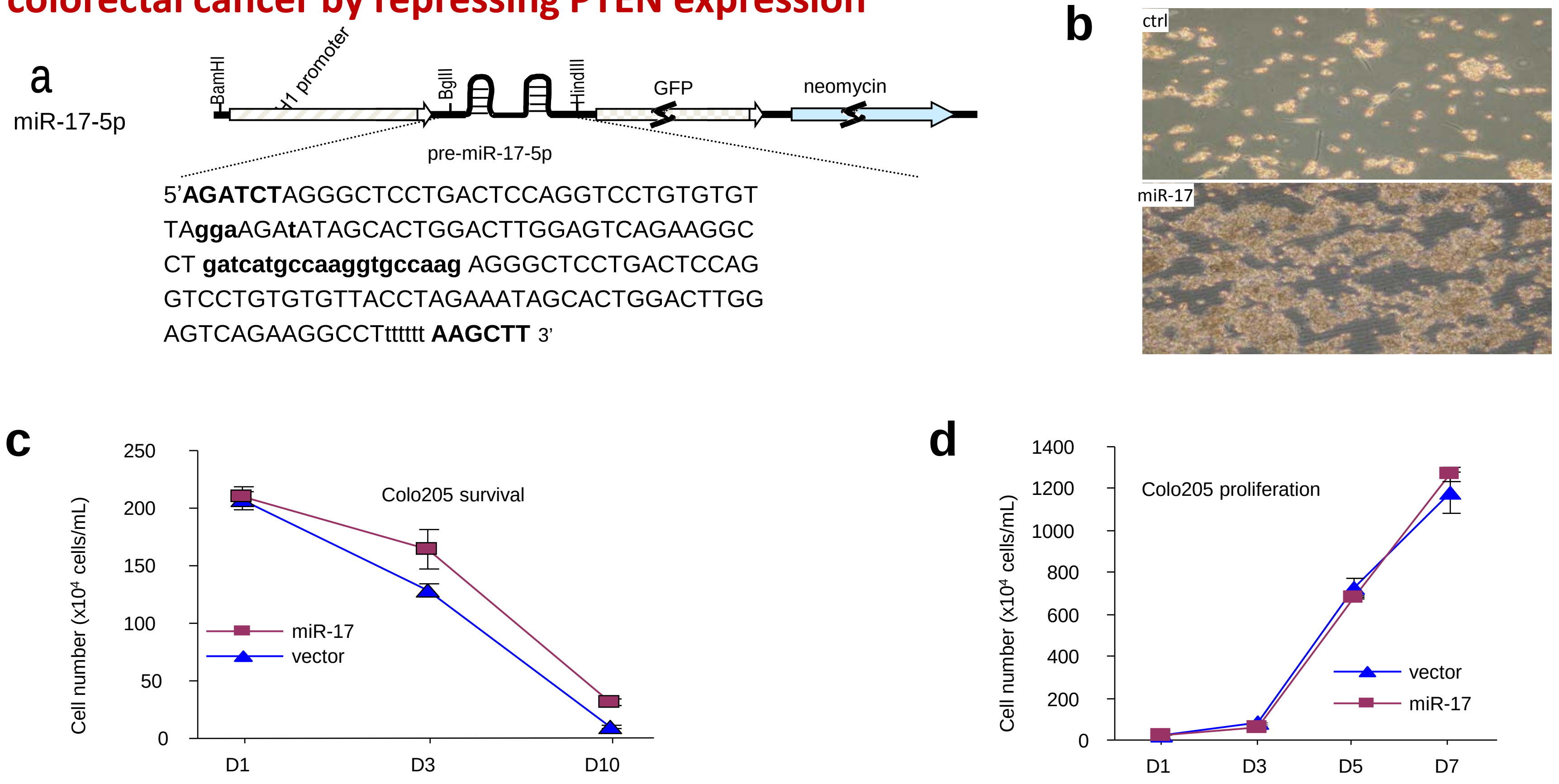

Supplementary Fig S1. Drug resistance of cells expressing miR-17. (a) Structure and sequence of miR-17 expression construct. (b) Colo205 cells transfected with miR-17 survival longer in the presence of drugs as compared with the control. (c-d) In the absence of drug, there was little difference in cell survival (c) and proliferation (d) in cells transfected with miR-17 or a control vector.

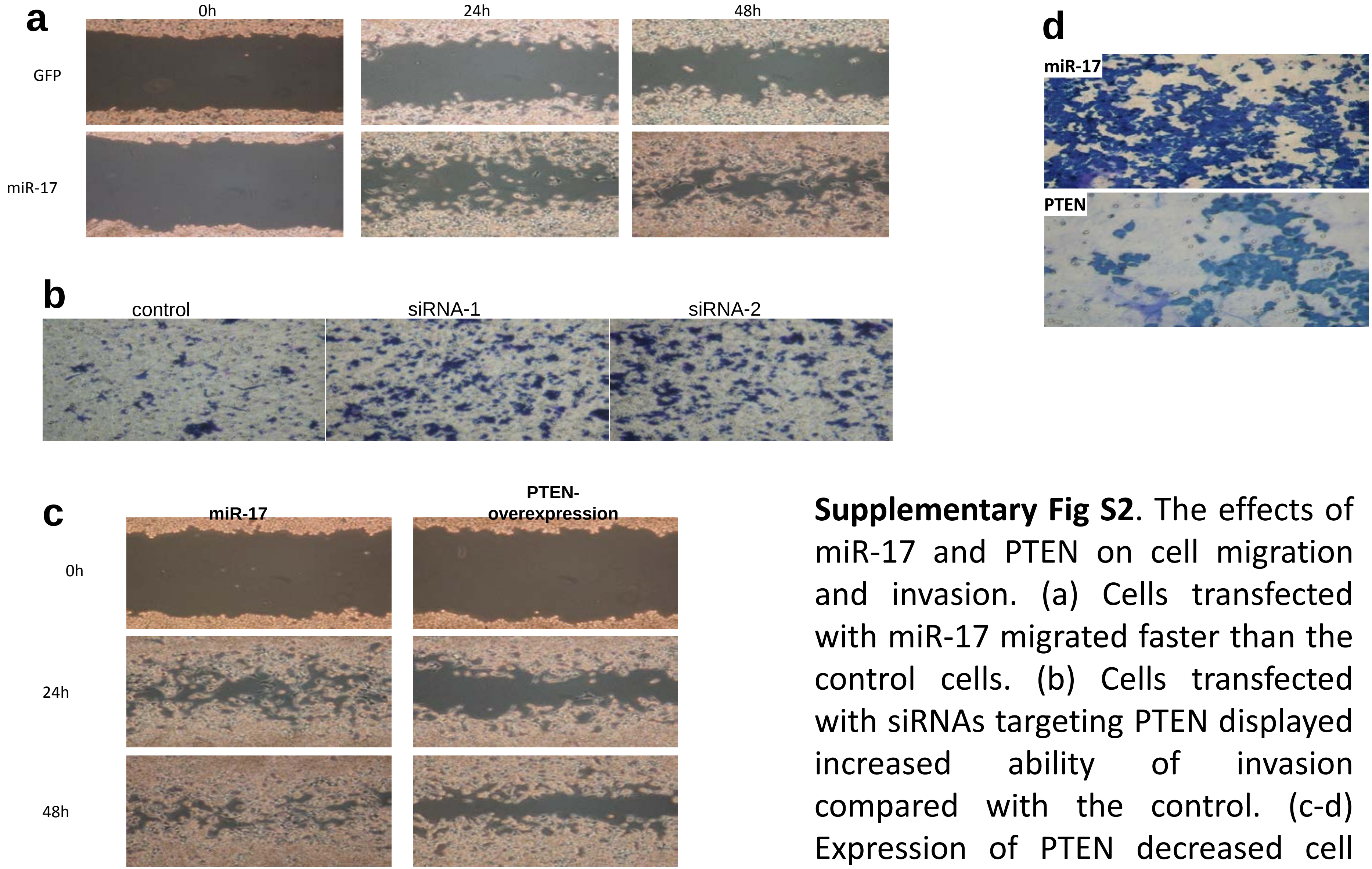

Supplementary Fig S2. The effects of miR-17 and PTEN on cell migration and invasion. (a) Cells transfected with miR-17 migrated faster than the control cells. (b) Cells transfected with siRNAs targeting PTEN displayed increased ability of invasion compared with the control. (c-d) Expression of PTEN decreased cell migration (c) and invasion (d) compared with the control.
