# Supplementary material for: MicroRNA-17-5p promotes chemotherapeutic drug resistance and tumour metastasis of colorectal cancer by repressing PTEN expression
Source: Oncotarget. 2014 Jan 19;5(10):2974–87. doi: 10.18632/oncotarget.1614 (PMC4102784; doi:10.18632/oncotarget.1614)
Supplement: Supplementary file 2 [file oncotarget-05-2974-s002.pdf]

MicroRNA-17-5p promotes chemotherapeutic drug resistance and tumour metastasis of colorectal cancer by repressing PTEN expression

Supplementary Table S1. Correlation between expression of mir-17-5p and clinicopathological features in 295 cases of colorectal cancer

			mir-17		
		All cases	Low expression	Overexpression	P Value
Sex	Male	153 (51.9)	110 (53.4)	43 (48.3)	0.423
	Female	142 (48.1)	96 (46.6)	46 (51.7)	
Age	<59	134 (45.5)	96 (46.6)	38 (42.7)	0.536
	≥59	161 (54.5)	110 (53.4)	51 (57.3)	
Tumor location	Colon	148 (49.8)	104 (50.5)	43 (48.3)	0.732
	Rectum	149 (50.2)	102 (49.5)	46 (51.7)	
Histological grade	G1	24 (8.1)	17 (8.3)	7 (7.9)	0.353
	G2	225 (76.3)	161 (78.2)	64 (71.9)	
	G3	46 (15.6)	28 (13.6)	18 (20.2)	
pT status	1	8 (2.7)	5 (2.4)	3 (3.4)	0.443
	2	41 (13.9)	30 (14.6)	11 (12.4)	
	3	241 (81.7)	166 (80.6)	75 (84.3)	
	4	5 (1.7)	5 (2.4)	0 (0)	
pN status	0	181 (61.4)	127 (61.7)	54 (60.7)	0.874
	1	114 (38.6)	79 (38.3)	35 (39.3)	
pM status	pM0	265 (89.8)	192 (93.2)	73 (82.0)	0.004
	pM1	30 (10.2)	14 (6.8)	16 (18.0)	
Clinical stage	I	32 (10.8)	22 (10.6)	10 (11.2)	0.030
	II	129 (43.4)	94 (45.2)	35 (39.3)	
	III	106 (35.7)	78 (37.5)	28 (31.5)	
	IV	30 (10.1)	14 (6.7)	16 (18.0)	
Chemotherapy	No	214 (72.5)	151 (73.3)	63 (70.8)	0.657
	Yes	81 (27.5)	55 (26.7)	26 (29.2)	

Supplementary Table S2. Univariate and multivariate analysis of different prognostic parameters in 214 colorectal cancer patients without chemotherapy

		Univariate analysis [a]			Multivariate analysis [b]	
	Variable	All cases	Mean survival (years)	p Value	HR (95% CI)	p Value
Sex	Male	108	6.492	0.816	1	0.887
	Female	106	6.434		0.963(0.572 to 1.620)	
Age	<58.8	86	6.707	0.139	1	0.085
	>58.8	128	6.292		1.632(0.935 to 2.850)	
Tumor location	Colon	104	6.715	0.153	1	0.046
	Rectum	110	6.228		1.754(1.011 to 3.045)	
Histological grade	G1-G2	190	6.580	0.053	1	0.226
	G3	24	5.500		1.538(0.766 to 3.045)	
pT status	T1-T2	43	6.949	0.289	1	0.855
	T3-T4	171	6.342		1.072(0.509 to2.256)	
pN status	N0	144	6.765	0.019	1	0.001
	N1	70	5.825		2.617(1.480 to 4.627)	
pM status	pM0	192	6.874	<0.001	1	<0.001
	pM1	22	2.859		11.537 (5.883 to 22.626)	
miR-17 expression	Low expression	151	6.740	0.023	1	0.075
	Overexpression	63	5.829		1.647 (0.951 to 2.853)	
